# Mechanism-Driven Green Extraction of Plant Polyphenols: From Molecular Interactions to Process Integration and Intelligent Optimization

**DOI:** 10.3390/plants15040596

**Published:** 2026-02-13

**Authors:** Shiwei Yuan, Wanru Zhao, Yongli Wang, He Dong, Kai Song, Dongfang Shi

**Affiliations:** 1 School of Life Science, Changchun Normal University, Changchun 130032, China; ccsfysw00@163.com (S.Y.); zhaowanrumei@163.com (W.Z.); mdonghe@163.com (H.D.); 2 Department of Civil, Environmental, & Construction Engineering, Texas Tech University, Lubbock, TX 79409, USA; aenguswang8@gmail.com; 3 Institute of Innovation Science and Technology, Changchun Normal University, Changchun 130032, China

**Keywords:** plant polyphenols, green extraction, deep eutectic solvents, synergistic effects, molecular interactions

## Abstract

Plant polyphenols are valuable secondary metabolites with significant bioactivities; however, their efficient extraction faces multiple challenges, including the structural complexity arising from their coexistence in free and bound forms within plant matrices, as well as their sensitivity to oxidation and heat. Although emerging green extraction technologies such as deep eutectic solvents, supercritical fluid extraction, and physical field enhancement show potential, current research largely remains method-oriented, lacking an in-depth understanding of the coupling mechanisms between molecular interactions and mass transfer processes. This review explicitly proposes a “mechanism-driven, synergistic integration” framework for the green extraction of plant polyphenols. By systematically analyzing the molecular basis of extractability and the complementarity among emerging technologies, this framework provides theoretical guidance and a practical blueprint for transitioning from empirical optimization to intelligent, synergistic system design. Specifically, it begins by systematically dissecting the structural characteristics of polyphenols and their interactions with cell wall components to clarify the molecular basis of extractability. Next, it critically reviews the mechanisms, advantages, and engineering bottlenecks of representative green technologies, with a focus on how synergistic integration strategies based on complementary mechanisms can overcome the limitations of single technologies to achieve higher extraction efficiency and selectivity. Furthermore, it evaluates the application of response surface methodology and artificial neural networks in process modeling. Finally, it highlights critical challenges such as industrial scale-up, sustainability assessment, and intelligent manufacturing. This review advocates a paradigm shift from optimizing single techniques toward designing intelligent, synergistic systems grounded in mechanistic insights.

## 1. Introduction

Plant polyphenols are a class of natural active substances characterized by phenolic hydroxyl groups, widely distributed in fruits, vegetables, tea, grains, legumes, and traditional Chinese medicines. They possess dual functional attributes, offering biological activities such as antioxidant, anti-inflammatory, and metabolic regulation effects, while also serving as functional ingredients in food packaging (e.g., pH-responsive indicators) [1,2,3]. With the growing global demand for natural health products, the application of polyphenols in functional foods and pharmaceuticals continues to expand [4,5,6].

However, the high-value utilization of polyphenols is fundamentally constrained by the trade-off between “extractability” and “stability retention.” This dilemma stems from the complex existence of polyphenols within the plant matrix and their inherent physicochemical sensitivity. Unlike simple chemical systems, plant extraction is a heterogeneous mass transfer process strictly constrained by the cellular architecture. Polyphenols not only exist in a free state within the vacuole but a significant proportion is also tightly bound to cell wall polysaccharides, proteins, and lignin via ester bonds, hydrogen bonds, and hydrophobic interactions, forming a “bound state” reservoir [7,8]. Furthermore, the active phenolic hydroxyl groups—the basis of their biological function—are fragile sites sensitive to heat, oxygen, and harsh solvent environments. Although traditional methods (e.g., heat reflux, organic solvent extraction) are simple, they often rely on high-intensity solvent input and prolonged processing, making it difficult to balance yield with activity retention, while also raising concerns regarding high energy consumption and solvent residues [9,10].

**Figure 1:**
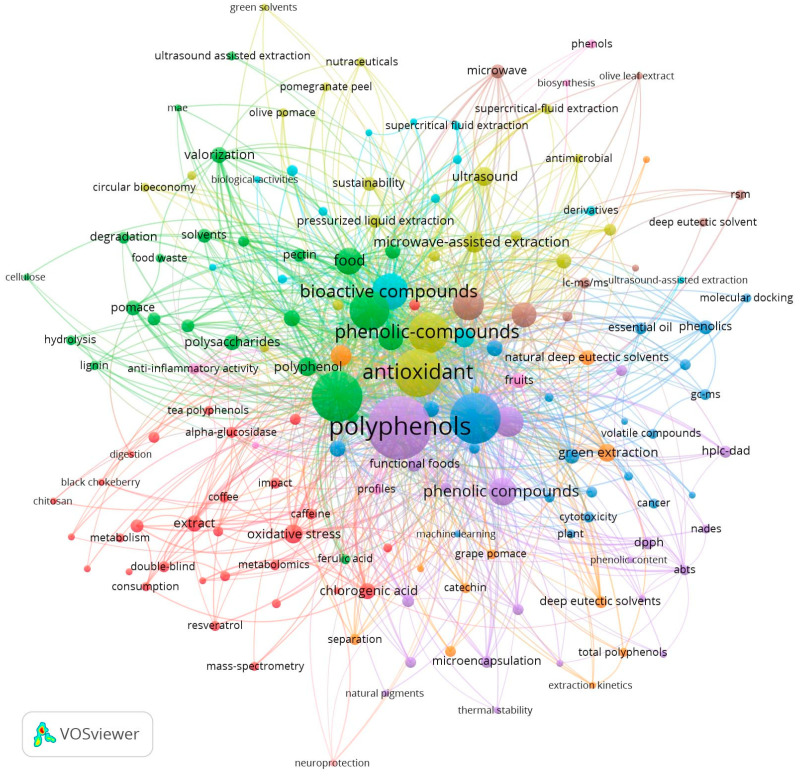
Recent trends in plant polyphenol extraction process innovation.

To address these challenges, emerging green extraction technologies have been developed. A keyword co-occurrence network analysis based on the Web of Science Core Collection (Figure 1) reveals that in the past decade, terms such as “ultrasound/microwave-assisted extraction,” “deep eutectic solvents (DESs),” and “supercritical fluid extraction (SFE)” have emerged as core clusters closely associated with “polyphenols” and “antioxidant activity”. This trend indicates a consensus on using physical fields to enhance mass transfer or designing novel green media to reduce energy consumption and solvent residues. Physical field technologies (e.g., ultrasound, microwave) directly disrupt cellular structures through mechanical, thermal, or electromagnetic effects, significantly shortening extraction times. Meanwhile, green solvent systems (e.g., DESs, SFE) provide highly selective dissolution environments through tunable physicochemical properties or molecular interactions. However, single technologies often face inherent performance boundaries: physical fields efficiently disrupt cell walls but struggle to improve intrinsic solvent selectivity, while green solvents offer molecular recognition potential but are often limited by high viscosity or low polarity.

Consequently, the research paradigm in this field is undergoing a profound shift from pursuing “better single technologies” to exploring “smarter technological integration.” Recent bibliometric analyses clearly show that terms like “synergistic extraction,” “integrated processes,” and “coupling mechanisms” are rapidly growing in relevance. This integration is not a simple superposition of technologies but a strategic combination based on a deep understanding of multi-scale interactions among polyphenols, the matrix, solvents, and energy fields. For instance, combining the selective dissolution of DESs with the cavitation effect of ultrasound (DES-UAE), or coupling the high permeability of supercritical fluids with the specific degradation of enzymes, has been proven to break the bottlenecks of single technologies [11,12,13]. While several recent reviews have cataloged the performance of diverse green technologies, the majority remain method-oriented, often presenting extraction results as independent data points without bridging the gap between molecular-level interactions and macroscopic efficiency [14,15]. Consequently, there is a critical need for an analytical framework that shifts the paradigm from simple technology comparison to intelligent technological synergy based on mechanistic understanding.

In light of this, this review aims to propose a “mechanism-driven, synergistic integration” framework for plant polyphenol extraction. We no longer view various technologies as competing choices but as modular tools within a functional library. This article first analyzes the molecular basis of polyphenol extractability and critical limiting steps. It then systematically reviews the core mechanisms of major green extraction technologies and their synergistic potential, focusing on the complementary principles between different technologies (e.g., solvent–physical field, physical field–physical field). Furthermore, we discuss how to achieve step changes in system performance through rational design, supported by recent advances in Response Surface Methodology (RSM) and Artificial Neural Networks (ANN). Finally, we outline the future of synergistic process integration and intelligent manufacturing, aiming to provide a theoretical basis for precise process control and industrial scale-up. The core argument of this review is that future breakthroughs in plant polyphenol extraction will depend not on the extreme optimization of a single method, but on intelligent technological synergy and system integration based on a deep understanding of molecular mechanisms.

## 2. Molecular Basis of Plant Polyphenol Extractability

The essence of plant polyphenol extraction is a process in which target molecules overcome the binding energy within the plant matrix and cross the solid–liquid interface into the solvent phase. Therefore, “extractability” is governed by the coupling of three key factors: the structural and solvation characteristics of the polyphenol molecules, their existence forms within plant tissues (free vs. bound states), and the mass transfer pathways and resistances determined by the cell wall architecture. Based on the principles above, and to clarify the common patterns of polyphenol extraction, this review will focus on seven representative classes of phenolic compounds—including flavonoids, phenolic acids, anthocyanins, and tannins. These groups were selected not only for their high abundance in agri-food matrices but, more importantly, because they encompass three distinct matrix-binding and mass-transfer models: (i) free-state solubilization, (ii) covalent-bond cleavage (e.g., cell-wall-bound phenolic acids), and (iii) macromolecular desorption (e.g., complex tannins). By dissecting the extraction mechanisms of these prototypical classes, this framework provides a universal theoretical basis that can be extended to other phenolics, such as coumarins and anthraquinones, which exhibit analogous thermodynamic and kinetic behaviors.

### 2.1. Structural Heterogeneity and Solvation Thermodynamics

Plant polyphenols are not uniform chemical entities but a heterogeneous collection of secondary metabolites sharing a phenylpropanoid backbone with complex modifications. Differences in hydroxyl substitution patterns, conjugated systems, and degrees of polymerization directly define the thermodynamic boundaries and kinetic behaviors during extraction.

Phenolic hydroxyl groups, as the core functional units, possess a distinct dual nature. They are key sites for forming hydrogen-bond networks with polar solvents, which promotes solvation under the “like dissolves like” principle. However, when these hydroxyl groups are located at ortho positions on the B-ring (e.g., catechins and chlorogenic acid), they also create oxidation- and heat-sensitive sites. Thus, molecular structure is both the target of extraction and a constraint on operating conditions (e.g., temperature, energy density). It is essential to emphasize that the extractability of polyphenols is fundamentally governed by their chemical speciation, such as whether they exist as free aglycones, glycosides, or complex esters. As detailed in Table 1, these variations significantly dictate the molecular weight ranges and polarity trends of each class. For instance, the presence of sugar moieties or organic acid acyl groups not only shifts the pKa and solubility but also dictates the dominant thermodynamic interactions with the plant matrix (e.g., hydrogen bonding vs. covalent linkages). Consequently, the primary rate-limiting steps transition from simple solubility-driven diffusion to complex chemical dissociation or macromolecular transfer barriers.

It must be clarified that while current green extraction technologies often yield complex mixtures rather than purified fractions, the fundamental mass-transfer resistances encountered during the process are highly class-specific. As shown in Table 1, the thermodynamic energy barrier for releasing vacuolar flavonoids (governed by intracellular diffusion) differs fundamentally from that of cell-wall-bound phenolic acids (governed by covalent bond dissociation). Recognizing these mechanistic disparities is not meant to imply downstream separation, but rather to provide a theoretical basis for selecting synergistic inputs—such as using enzymatic hydrolysis to target ester-linked phenolic acids or acoustic cavitation to enhance the desorption of high-molecular-weight tannins. This mechanism-driven differentiation is the prerequisite for moving beyond trial-and-error toward strategic process integration.

Furthermore, it is noteworthy that the physicochemical behavior of crude extracts (e.g., hygroscopicity or stability) is often a collective manifestation of both target polyphenols and co-extracted matrix components (such as residual sugars, organic acids, or proteins). Therefore, the “Common Issues” highlighted in Table 1 represent the intrinsic challenges associated with the polyphenol molecules themselves, while practical process design must account for these multicomponent interactions to avoid misleading interpretations of extract properties in real-world scenarios.

### 2.2. Matrix Resistance: From Free to Bound States

**Figure 2:**
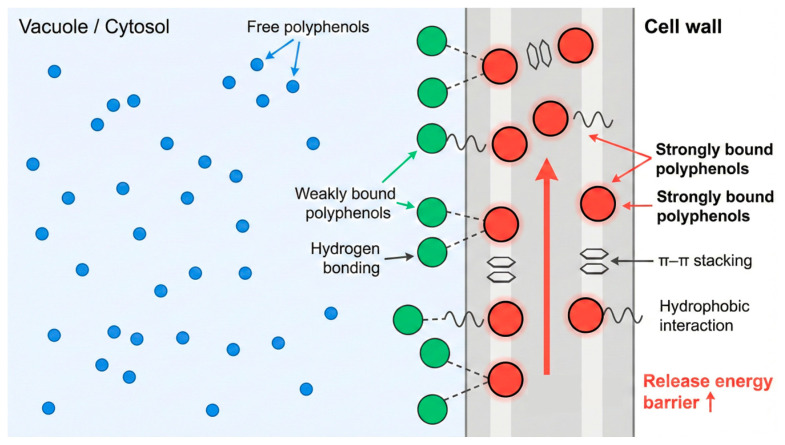
Schematic representation of the existence forms of plant polyphenols in the plant matrix and their molecular interactions.

In plant tissues, polyphenols do not exist entirely in a free state. As illustrated in Figure 2, while free polyphenols are located in the cytosol or vacuoles and are relatively easy to release with conventional solvents, a significant proportion exists as “bound phenolics.” These compounds are distributed within the cell wall or intercellular spaces, tightly bound to macromolecules such as polysaccharides, proteins, and lignin via covalent or non-covalent interactions. This phenomenon is considered a root cause of limited extraction efficiency and the variability observed across different extraction techniques [27].

From a molecular perspective, the three-dimensional network composed of cellulose, hemicellulose, and pectin in the cell wall provides abundant binding sites: phenolic hydroxyl groups can form stable hydrogen bonds with polysaccharide hydroxyls, while aromatic rings may interact with lignin components through hydrophobic interactions or π–π stacking [28]. It is the superposition of these multiple forces that makes bound polyphenols difficult to fully release under conventional extraction conditions. Consequently, relying solely on solvent polarity regulation is often insufficient; auxiliary strategies capable of disrupting cell wall structures or specifically cleaving chemical bonds—such as physical field enhancement or enzymatic hydrolysis—must be introduced to overcome this “binding energy barrier.”

### 2.3. Critical Rate-Limiting Steps in Extraction

Physically, the “extractability” of polyphenols can be summarized as a mass transfer landscape where target molecules must overcome matrix binding energies and successfully cross the phase interface into the solvent. Based on the extraction behaviors detailed in Table 1, this process is generally constrained by three critical bottlenecks. The first is desorption or dissolution limitation, which occurs when the interactions between polyphenols and the plant matrix (e.g., hydrogen bonding or hydrophobic effects) prevail over solvent–solute interactions; under such conditions, merely increasing solvent volume or extraction time is often ineffective. The second constraint is internal diffusion limitation, arising from intact cellular structures and tortuous pathways that significantly hinder the migration of molecules from the interior of the matrix to the surface. Finally, external mass transfer resistance becomes a defining factor, particularly in high-viscosity systems (such as those involving certain DESs) or under boundary-layer control, where the efficient transport of polyphenols from the matrix surface into the bulk solvent phase is impeded.

## 3. Solvents: Tools for Desorption and Dissolution

Traditional plant polyphenol extraction has long relied on water, organic solvents, and their mixtures, operating on the principle of “like dissolves like” to match the polarity of target compounds. While aqueous organic solvents (e.g., ethanol, methanol) are efficient for crude extraction, their effectiveness stems largely from non-specific solvation. They facilitate desorption but lack the ability to “selectively recognize” specific functional groups. Consequently, when the interactions (e.g., hydrogen bonding, π–π stacking) between polyphenols and the plant matrix are stronger than solvent–polyphenol interactions, simply increasing solvent volume fails to break the “bound state.”

With the deepening of green chemistry, the definition of green solvents has expanded from merely “low toxicity” to “designable functionality.” In polyphenol extraction, research has evolved along two distinct paths: DESs, representing “interaction-designable media,” and SFE, representing “property-tunable media.” As summarized in Table 2, which compiles studies on various extraction technologies including DESs and SFE, each technology exhibits unique mechanistic niches: for instance, SFE is particularly suitable for low-molecular-weight aglycones due to its tunable non-polar nature, while enzymatic-assisted extraction (EAE) is indispensable for releasing phenolic acids covalently anchored to the cell wall. Understanding such technology-speciation synergies enables a more precise approach to maximizing the recovery of intact, bioactive molecular forms while minimizing the generation of non-target by-products.

### 3.1. Deep Eutectic Solvents (DESs)

**Figure 3:**
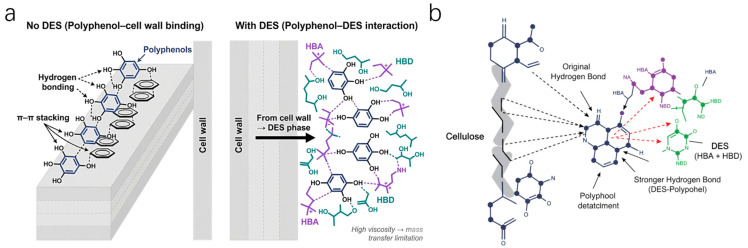
DES-assisted extraction of polyphenols: hydrogen-bond competition. (**a**) Representative components for DES formulation. (**b**) Competitive hydrogen bonding with polyphenolic hydroxyl groups, weakening polyphenol–matrix interactions and promoting release.

DESs are typically composed of a hydrogen bond acceptor (e.g., quaternary ammonium salts) and a hydrogen bond donor (e.g., alcohols, acids) mixed at a specific molar ratio to form a eutectic mixture through hydrogen bonding. Their core mechanism lies in their hydrogen bond formation capability. Figure 3a illustrates the chemical diversity of common hydrogen bond acceptor and donor components. Beyond their structural properties, the extraction superiority of DESs stems from a hydrogen bond competition mechanism (as depicted in Figure 3b). The designed hydrogen bond network of DESs effectively disrupts the intrinsic interactions between polyphenols and the plant cell wall, significantly lowering the energy barrier for solute release [52].

From a molecular thermodynamic perspective, DES is not merely a conventional solvent; it acts as an interaction-tunable medium. The high density of hydrogen bond acceptors and donors within the DESs creates a competitive environment that disrupts the pre-existing polyphenol-matrix complexes. By establishing stronger intermolecular hydrogen bonds with the phenolic hydroxyl groups, the DESs effectively lower the desorption energy barrier, enabling the transition of bound phenolics into the liquid phase.

Compared to traditional solvents, DESs offer distinct advantages: they have negligible vapor pressure, are biodegradable, and exhibit excellent thermal and chemical stability [53]. Particularly noteworthy is that by adjusting the combination of hydrogen bond acceptors and donors, DESs can be “tailored” to match the specific polarity of target polyphenols (for example, Type III DESs composed of choline chloride and carboxylic acids/polyols are the most widely used). However, the inherent engineering bottleneck of DESs lies in their high viscosity. This significantly increases internal diffusion resistance, meaning that their “strong interaction advantage” often cannot translate into a macroscopic yield advantage. Current research highlights the tunable nature of DESs in extracting diverse phenolics from edible oils and agri-food residues [54,55]. However, the selective solvation energy of DESs often requires synergistic physical assistance to overcome the matrix resistance effectively [56,57]. In summary, as an emerging green solvent, DESs are generally most suitable for the extraction of polar polyphenols where environmental friendliness is a priority. Its key limitations include high intrinsic viscosity (which restricts mass transfer) and subsequent target compound separation and solvent recovery, thereby hindering its large-scale industrial implementation.

### 3.2. Supercritical CO_2_ Extraction (SFE)

**Figure 4:**
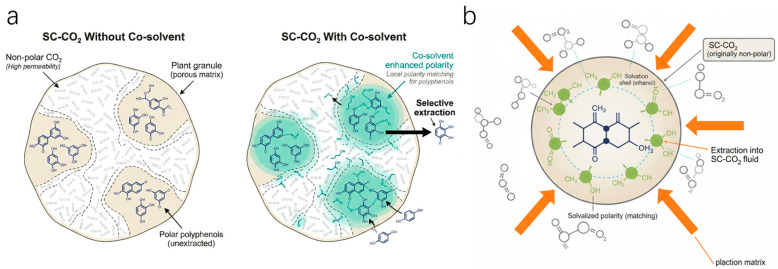
Supercritical CO_2_ extraction of polyphenols: matrix transport and polarity modulation. (**a**) Diffusion and penetration of supercritical CO_2_ in porous plant matrices. (**b**) Polarity modulation by an entrainer to enhance polyphenol solubility and selectivity.

The advantage of SFE stems from its unique dual characteristics: liquid-like density grants it solvation capability, while gas-like viscosity ensures high diffusivity and permeability [58]. As shown in Figure 4a, supercritical carbon dioxide maintains liquid-like density while simultaneously possessing gas-like low viscosity and zero surface tension, enabling it to function as a highly permeable medium that can easily penetrate the intricate pore structures of plant matrices. However, to compensate for its polarity mismatch with polyphenolic compounds, the introduction of entrainers is crucial. Figure 4 visually illustrates this mechanism: polar entrainers achieve a “localized polarity matching” effect by forming a concentrated solvation shell around the target molecules. This dual-action mechanism—macro-scale penetration (Figure 4a) coupled with micro-scale solvation (Figure 4b)—allows for the efficient and selective extraction of heat-sensitive polyphenolic compounds without the risk of solvent residues. Overall, supercritical CO_2_ provides rapid transport, whereas polar entrainers (e.g., ethanol) create a transient solvated micro-environment that improves polyphenol solubility and selectivity, enabling residue-free extraction under mild thermal conditions.

However, the main limitation of this technology lies in its typically higher extraction cost for highly polar polyphenols compared to conventional solvent extraction. It is therefore more suitable for applications targeting high-value fractions, especially where solvent-free products are required—such as in the extraction of lipophilic or moderately polar, heat-sensitive polyphenols. Furthermore, high equipment costs due to the need for high-pressure systems and the relative complexity of the process also constrain its widespread adoption.

In summary, traditional solvents, DESs, and SFE should not be viewed merely as “advanced replacing backward” but as complementary tools with distinct mechanisms. Traditional solvents offer low viscosity for rapid crude extraction but lack selectivity; DESs offer designable selectivity but suffer from viscosity issues; and SFE offers high permeability and purity but is limited by polarity and cost. Therefore, the optimization direction lies in integrating these solvents into a cohesive “solvent–process–separation” system.

## 4. Physical Fields and Enzymes: Tools for Structural Modification and Mass Transfer

**Figure 5:**
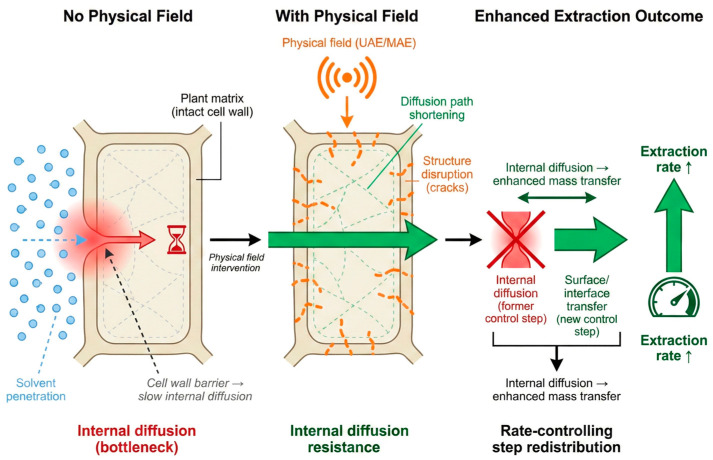
Mechanism overview of “rate-limiting step redistribution” in physical field-assisted extraction.

Unlike solvent systems that primarily modulate molecular interactions, the core role of physical field enhancement and enzymatic hydrolysis is to reduce the structural and diffusional resistances that polyphenols must overcome to migrate from the matrix to the solvent phase (Figure 5).

In most extraction systems, solvent molecules must penetrate the cell wall, diffuse internally, and then transport across the interface. Physical fields do not merely increase solubility; rather, by disrupting tissue structures and altering energy distribution, they effectively reallocate the rate-controlling steps. For instance, they can shift the process from being “internal diffusion-controlled” (a bottleneck due to intact cell walls) to “surface transfer-controlled,” thereby allowing the solvent’s potential to be fully realized.

### 4.1. Ultrasound-Assisted Extraction (UAE)

**Figure 6:**
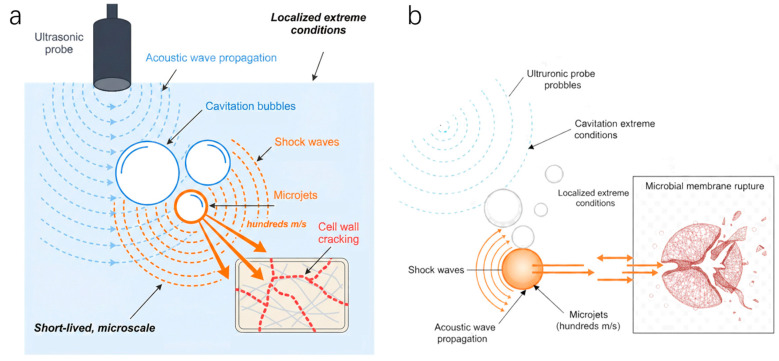
Ultrasound-assisted extraction of polyphenols: cavitation effects and structural disruption. (**a**) Acoustic cavitation bubble growth and collapse, generating micro-jets and shock waves. (**b**) Cell-wall pitting and cracking, reducing mass-transfer resistance and accelerating extraction.

UAE is widely used due to its maturity and ease of coupling. Its fundamental mechanism is acoustic cavitation: the rapid formation and collapse of microbubbles generate localized extreme temperatures (~5000 K), high pressures (~1000 atm), and high-speed microjets. The enhancement arises primarily from a multiscale mechanical action. As shown in Figure 6a, acoustic waves induce cavitation bubble growth and collapse, generating micro-jets and shock waves. As shown in Figure 6b, the resulting shear forces cause surface pitting and cracking of the cell wall. This structural disruption reduces intraparticle diffusion resistance and accelerates solvent renewal within the matrix. Consequently, the rate-limiting step shifts from internal diffusion toward surface convection and interfacial mass transfer [59].

Beyond the simple cell-wall rupture, the micro-jets and shock waves from acoustic cavitation create a ‘pitting effect’ within the plant matrix. Repeated compression–expansion promotes solvent renewal within the tissue and accelerates the efflux of solubilized polyphenols. This mechanical action effectively reallocates the rate-limiting step from internal diffusion to surface mass transfer.

However, UAE follows a non-linear efficiency curve. Literature indicates that polyphenol yields often exhibit a trend of “increasing first and then decreasing” with rising power or time [60,61]. This is due to a competition between two mechanisms: while cavitation enhances cell disruption, excessive energy input triggers free radical generation and mechanical shearing, leading to the degradation of sensitive polyphenolic structures [62]. This explains why optimal windows vary significantly across raw materials. For example, while moderate conditions (200 W, 30 min) are sufficient for hemp cake polyphenols [40], denser materials like walnut green husks require lower power (160 W) but longer duration (60 min) to balance release with stability [63]. UAE is most suitable for laboratory or small-to-medium scale applications, enabling rapid and efficient preliminary extraction of heat-sensitive polyphenols. It is particularly effective for easily processed materials such as plant leaves and fruit pulp. The key limitations of this technology include uneven distribution of ultrasonic energy during scale-up, which reduces efficiency, potential degradation of polyphenols under prolonged exposure, and continued reliance on organic solvents.

### 4.2. Microwave-Assisted Extraction (MAE)

**Figure 7:**
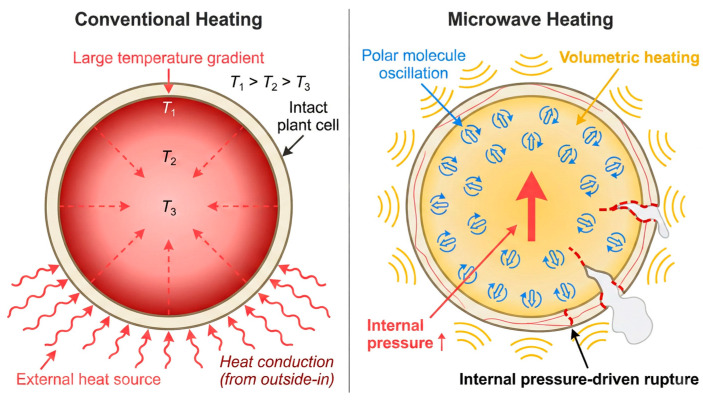
Schematic of volumetric heating and internal pressure-driven rupture in MAE.

MAE operates primarily through the interaction between electromagnetic fields and polar molecules, such as water and intracellular fluids. Microwaves penetrate plant tissues, causing polar molecules to rotate and collide billions of times per second, thereby generating rapid and uniform volumetric heating within the material. This “inside-out” heating mechanism fundamentally differs from conventional conductive heating, as it rapidly increases intracellular pressure, creating a “micro-explosion” effect that ruptures cell walls from within and increases matrix porosity. This process establishes efficient transport channels for the release of target compounds such as polyphenols (Figure 7) [64,65].

Due to these characteristics, MAE offers a significant advantage in extraction speed, particularly for materials with dense structures and poor solvent permeability, often achieving in minutes what traditional methods require hours to accomplish [42]. However, the rapid heating also carries the risk of “thermal runaway,” where localized temperatures may exceed the thermal stability threshold of polyphenols, leading to degradation. Therefore, MAE is best employed as a rapid “pretreatment module” to efficiently disrupt cellular structures and create a porous system before a milder extraction phase.

In summary, this technique is most suitable for the rapid extraction of polar polyphenols from plant materials, demonstrating particularly high efficiency for samples with high moisture content or rigid cellular structures, such as seeds and bark. Its main limitations include poor effectiveness for non-polar polyphenols and the tendency for localized overheating to degrade heat-sensitive active components.

### 4.3. High Hydrostatic Pressure Extraction (HHPE)

HHPE utilizes ultra-high isotropic hydraulic pressure (typically 100–1000 MPa) to mechanically disrupt the plant matrix. The pressure compresses air vacuoles within the cell, causing irreversible mechanical damage to cell walls and membranes, thereby releasing polyphenols. Crucially, this process involves minimal temperature rise (usually <30 °C), making it a highly effective “cold extraction” technology that maximally preserves the bioactivity and structural integrity of heat-sensitive polyphenols [66]. HHPE is most suitable for extracting thermolabile and oxidation-prone polyphenols from liquid or high-moisture samples such as fruit juices and berry pulps, maximizing the preservation of their native bioactivity. Its key limitation lies in its restricted applicability to specific material types, exhibiting lower efficiency with dry or rigid matrices.

### 4.4. Enzyme-Assisted Extraction (EAE)

EAE targets the chemical barrier of the cell wall. By selecting specific enzymes (e.g., cellulase, pectinase), EAE specifically hydrolyzes the polysaccharide network (cellulose, pectin) that binds polyphenols. This biological specificity allows for the release of “bound phenolics” that are inaccessible to solvents alone, without damaging the target compounds. The mild conditions (40–60 °C, pH 4–6) further ensure the retention of biological activity. This method is particularly well-suited for processing raw materials with complex cell wall structures, such as those high in pectin and cellulose. It selectively releases embedded polyphenols under mild conditions, effectively preventing oxidation. However, its key limitations include the high cost of enzymatic preparations, lengthy reaction times, and the strict control required over process conditions such as pH and temperature.

### 4.5. Pulsed Electric Fields (PEF) and High Voltage Electrical Discharge (HVED)

The mechanism of PEF is rooted in “electroporation” [67]. High-intensity, short-duration electric pulses induce polarization in the cell membrane lipid bilayer, forming transient or permanent hydrophilic pores. This non-thermal process (temperature rise <5 °C) selectively destroys the semi-permeable barrier, facilitating polyphenol release via diffusion. Best suited for energy-efficient, rapid, and non-thermal extraction from liquid or high-moisture plant tissues (e.g., fruit pulps and tea suspensions), where membrane permeabilization readily promotes solute diffusion. Limitations include reduced effectiveness in dry/low-moisture matrices and strong dependence on sample conductivity and treatment-chamber design.

In contrast, HVED involves a high-intensity energy deposition process accompanied by shock waves, UV radiation, and localized hotspots [68]. Best suited for hard or structurally complex matrices (e.g., grape seeds and fruit peel residues), where shock waves and discharge-induced effects can rapidly disrupt tissues and promote release. Limitations include localized heating and radical formation, which may accelerate oxidative degradation of sensitive polyphenols. It is worth noting that integrating these electrical technologies with DESs presents a challenge. Since DESs are highly conductive ionic systems, they can interfere with electric field distribution or cause energy dissipation and short circuits, making direct coupling difficult compared to non-conductive solvent systems.

## 5. Synergistic Integration of Extraction Technologies via Mechanistic Complementarity

The advantage of physical field technologies, such as UAE and MAE, lies in their ability to directly intervene in plant tissue structures and mass transfer pathways. However, their enhancement effects have clear boundaries. First, physical fields typically cannot alter the intrinsic “polyphenol–solvent” thermodynamic interactions; if the solvent lacks sufficient selectivity for the target polyphenol, enhancing cell wall disruption may simply increase the co-extraction of impurities (e.g., sugars, proteins). Second, physical field enhancement is accompanied by energy input. Once the energy exceeds the threshold required for structural disruption, the excess energy is more likely to induce polyphenol oxidation or degradation rather than a proportional increase in yield.

**Figure 8:**
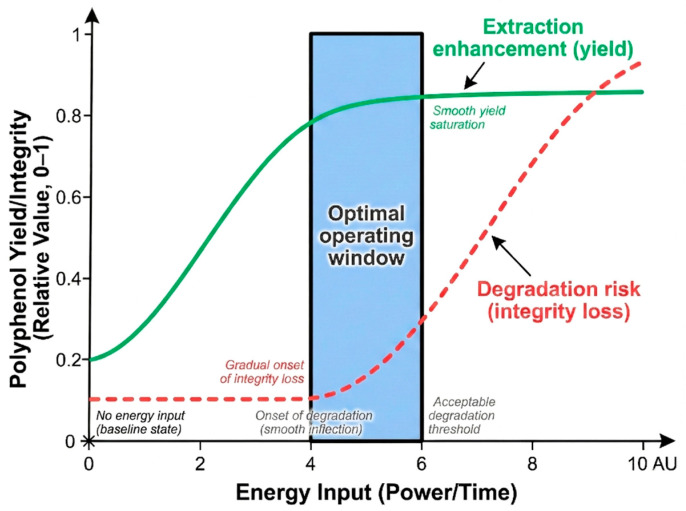
Schematic of the competitive relationship between extraction enhancement and compound degradation, defining the optimal operating window.

Therefore, the rational role of physical fields should be viewed as a “synergistic module” within the solvent system. By redistributing the rate-limiting steps in the “disruption–diffusion–dissolution/desorption” chain, they amplify the effectiveness of green solvents. This “enhancement–degradation” competition and the optimal operating window are summarized in Figure 8.

Crucially, synergy relies not on stacking intensities but on mechanistic complementarity: the solvent side enhances desorption and selectivity via polarity or hydrogen bonding networks, while the physical field side reduces internal mass transfer resistance by disrupting cell walls. When matched correctly, the rate-controlling step of the system shifts favorably from “internal diffusion-limited” to “dissolution/desorption-limited.” Table 3 summarizes representative synergistic strategies (e.g., DES–UAE, SFE–UAE).

### 5.1. Deep Eutectic Solvents Coupled with Ultrasound (DES-UAE)

**Figure 9:**
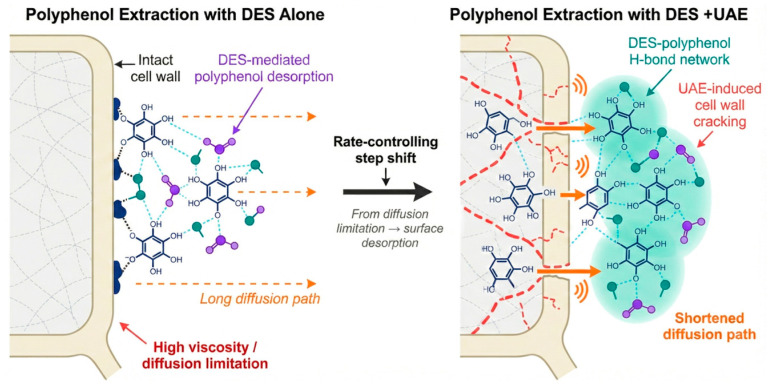
Schematic of the molecular mass transfer coupling mechanism in DES–UAE synergy.

The combination of green solvents and physical fields is the most representative synergistic strategy. Taking DES–UAE as an example, the mechanism is more than “DES dissolving + UAE breaking.” DESs enhance desorption and selective dissolution through hydrogen bond networks, but their high viscosity significantly increases internal diffusion resistance. UAE addresses this specific bottleneck: acoustic cavitation and mechanical shear disrupt boundary layers and enhance mixing, improving DES fluidity and penetration into the plant matrix [69]. Simultaneously, ultrasonic perturbation may facilitate the dynamic rearrangement of the DES–polyphenol hydrogen bond network, supporting solute release and transport. The “molecular mass transfer” coupling mechanism is illustrated in Figure 9.

However, this strategy has boundaries: excessive ultrasonic intensity can generate free radicals that may attack polyphenols, negating the selectivity advantage of the DES. Thus, the goal is not to maximize intensity but to find an operating window where the diffusion barrier is broken without compromising molecular integrity.

### 5.2. Supercritical CO_2_ Coupled with Ultrasound (SFE-UAE)

The integration of UAE with SFE creates a synergistic physical environment. The cavitation effect of ultrasound perturbs the boundary layer of the supercritical fluid, reducing mass transfer resistance and enhancing fluid convection and phase contact, thereby alleviating the diffusion limitation of SFE in complex matrices [70]. Conversely, the unique physical properties of the supercritical fluid can influence cavitation behavior, potentially enhancing the range of acoustic action [71]. Studies have shown that introducing ultrasound can reduce the required operating pressure of SFE by 10–30% while maintaining extraction efficiency [72]. This suggests that SFE-UAE is particularly suitable for high-value, heat-sensitive polyphenols where minimizing pressure and temperature is critical.

### 5.3. Microwave Coupled with Ultrasound (MAE-UAE)

Beyond solvent–field synergy, the coupling of different physical fields (e.g., UAE–MAE) is also gaining attention. However, unlike the former, coupling two physical fields risks “repetitive enhancement” if clear roles are not defined. Both ultrasound and microwaves disrupt cell structures, but their pathways differ: ultrasound relies on mechanical cavitation (local shear/cracks), while microwaves rely on volumetric heating (internal pressure).

**Figure 10:**
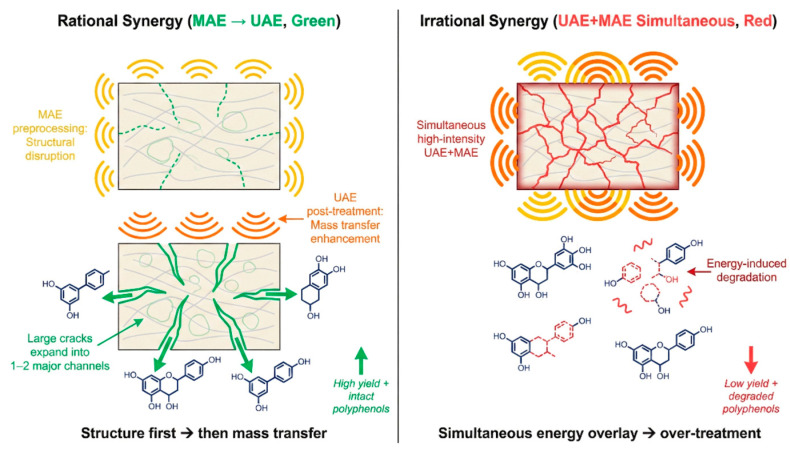
Schematic comparison of “Complementary vs. Repetitive” pathways in physical field synergy.

If combined irrationally (e.g., simultaneous high intensity), the energy inputs may overlap to trigger rapid degradation. A “rational synergy” typically involves a sequential approach: using MAE as a short-time pre-treatment to rapidly disrupt dense tissues and increase porosity, followed by milder UAE to enhance mass transfer. This “structure first, then transfer” strategy avoids the thermal degradation risks of prolonged microwave exposure while leveraging the diffusion enhancement of ultrasound [73]. The difference between complementary and repetitive pathways is visually demonstrated in Figure 10.

### 5.4. Enzyme Coupled with Ultrasound (EAE-UAE)

EAE-UAE represents a synergy between “biological specificity” and “physical mechanics.” Enzymes (e.g., cellulase, pectinase) specifically hydrolyze the chemical bonds of cell wall polysaccharides, structurally weakening the network integrity (“chemical softening”). On this basis, the mechanical forces generated by ultrasonic cavitation can more efficiently disintegrate the already weakened cell walls (“physical disintegration”) [74]. Furthermore, ultrasound can enhance enzyme–substrate contact through micro-stirring effects, reducing external diffusion limitations. This sequential or synchronous action achieves deep deconstruction of the cell wall barrier.

### 5.5. High Hydrostatic Pressure Coupled with Ultrasound (HHPE-UAE)

The combination of HHPE and UAE acts across multiple scales. High hydrostatic pressure pre-treatment places cell membranes and walls in a “pre-strained” state, reducing their mechanical strength. Applying ultrasound in this state induces more intense and focused cavitation events, effectively puncturing the “softened” structures. Additionally, the high-pressure environment can suppress the local thermal effects and vaporization associated with cavitation, making the process more controllable. This multi-scale synergy—from macroscopic pressure to microscopic cavitation—allows for efficient extraction at lower temperatures, effectively preserving heat-sensitive polyphenols [75].

In summary, synergy and integration in polyphenol extraction must be rationally designed with mechanistic constraints: first, identify the structural shortcoming of a single technology (e.g., DESs viscosity, SFE polarity, or degradation risks of physical fields); second, select a complementary technology that specifically targets that shortcoming; and finally, verify the favorable shift in the rate-controlling step within the “enhancement–degradation” framework (Figure 8).

The selection of green extraction technologies should not be empirical but rather dictated by the molecular characteristics of the target polyphenols and their existing forms within the plant matrix. To provide a practical roadmap for researchers, the correlation between polyphenol structural features, primary extraction barriers, and the most suitable synergistic strategies is integrated in Table 3. This comprehensive summary highlights how mechanistic complementarity—such as coupling designable solvent solvation with physical-field-induced structural disruption—can be strategically deployed to overcome specific rate-limiting steps, thereby achieving an optimal balance between yield, selectivity, and bioactivity retention.

**Table 3:** Strategic selection of synergistic extraction systems based on the physicochemical characteristics and matrix-binding modes of polyphenol classes.

Extraction Technique	Phenolics Suitable for Extraction	Key Structural Features & Limitations	Primary Extraction Barrier (Rate-Limiting Step)	Synergistic Mechanism (Mechanism Complementarity)	Ref.
DES-UAE	Flavonoids (e.g., Rutin, Quercetin)	Medium-high polarity; low solubility in single solvents.	Dissolution & Diffusion: Interaction between polyphenols and matrix.	DES provides a designable H-bond network to promote desorption; UAE cavitation reduces DES viscosity to enhance mass transfer.	[76]
HHPE-UAE	Phenolic acids, flavonoids, coumarins.	High water solubility; extreme pH and heat sensitivity.	Stability Retention: Degradation under high-energy or thermal conditions.	HHPE achieves high-efficiency “cold extraction” at <30 °C; subsequent UAE focuses on rupturing pre-strained cell structures.	[77]
EAE-UAE	Flavanols	Small molecules; often ester-bonded to cell wall polysaccharides.	Dissociation & Release: Chemical covalent bonds and matrix entrapment.	Enzymes (e.g., cellulase) specifically cleave covalent bonds; UAE enhances enzyme–substrate contact and speeds up leaching.	[78]
MAE-UAE	Tannins (Condensed & Hydrolyzable)	High MW; complex multi-point H-bonding; increases extract viscosity.	Macromolecule Desorption: Large molecular size and tortuous diffusion pathways.	MAE generates volumetric heating and internal pressure to rupture dense tissues; UAE improves the diffusion of large molecules into the bulk phase.	[79]
SFE-UAE	Low-polarity Phenolics (e.g., Stilbenes)	Low solubility in polar solvents; requires high purity.	Solvent Selectivity: Difficulty in balancing yield and fractionation purity.	SC-CO_2_ offers high permeability and selective dissolution; UAE perturbs the fluid boundary layer to reduce mass transfer resistance.	[71]

## 6. Process Optimization and Intelligent Modeling

### 6.1. Optimization Needs in Multi-Variable Coupling Contexts: From Single-Point to Operating Windows

**Figure 11:**
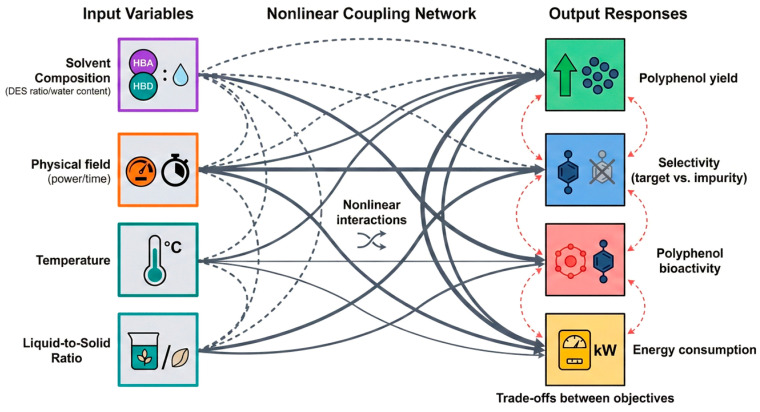
Schematic of multi-variable coupling and non-linear responses in synergistic extraction systems.

As discussed above, plant polyphenol extraction has evolved from a linear process dominated by a single factor to a coupled system driven by multiple factors. Variables such as solvent composition, physical field intensity, temperature, time, and solid-to-liquid ratio interact with each other. Especially with the introduction of synergistic technologies (e.g., DES–UAE, multi-physical fields), significant non-linear relationships and interaction effects exist among these variables. As shown in Figure 11, input variables collectively influence output responses (yield, selectivity/purity, bioactivity retention, and energy consumption) through a multi-layer coupling mechanism. Consequently, “empirical tuning” is no longer sufficient to find stable optimal solutions.

Furthermore, many studies indicate that responses often exhibit a “rise and then fall” trend with increasing power or temperature, suggesting the existence of a distinct optimal operating window. This window shifts depending on the raw material, solvent system, and synergistic mode. Therefore, the goal of optimization must expand from finding a single valid condition to defining a robust operational range under multiple constraints to support process scale-up. As demonstrated in Table 4, these parameters should not be viewed as isolated variables but as mechanistic tools to overcome specific molecular barriers. Optimization should be mechanism-driven and conditional on polyphenol speciation. Temperature is tuned not only for solubility but also to supply activation energy for releasing ester-linked phenolics. Solvent polarity is adjusted to match different glycosylation patterns. This “parameter-to-mechanism” mapping shifts optimization from empirical tuning to defining a robust operating window.

### 6.2. Response Surface Methodology (RSM): A Tool for Statistical Optimization Under Mechanistic Constraints

Response Surface Methodology (RSM) remains one of the most widely used statistical optimization tools in plant polyphenol extraction. Its core advantage lies in establishing a quadratic polynomial model with a relatively small number of experimental points, allowing for the evaluation of main factor effects and interactions within a defined experimental space to obtain an interpretable local optimum [90]. Compared to single-factor experiments, RSM systematically characterizes how variable interactions affect the response, offering clear engineering value for process screening and window positioning.

However, the applicability of RSM is strongly constrained by the experimental design space and variable selection. Different raw materials often show significant differences in optimal conditions, reflecting how plant structural features and polyphenol binding states influence the rate-controlling steps. Therefore, within a mechanism-driven framework, the rational role of RSM is to identify key variables and quantify interaction effects to determine operable windows, rather than extrapolating without constraints to extreme conditions that may lack physical meaning. Table 5 summarizes representative cases where RSM and ANN were used to handle nonlinear behavior in synergistic extraction. Crucially, these models are not merely statistical curve-fitting exercises; they aim to capture response surfaces linked to chemical speciation (e.g., aglycones, glycosides, and bound phenolic acids) under changing physical and chemical inputs. By mapping the interaction effects (e.g., between DESs’ polarity and ultrasonic cavitation thresholds), these modeling approaches provide a more faithful predictive framework than single-factor empirical designs.

### 6.3. Artificial Neural Networks (ANN): A Predictive Tool for Non-Linear and Complex Coupling

**Figure 12:**
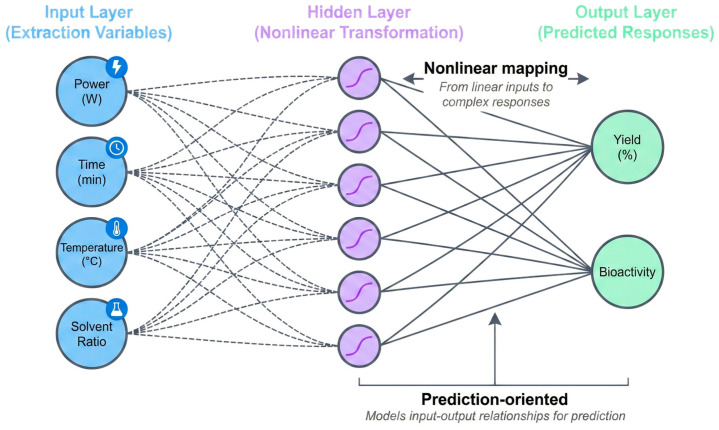
Schematic structure of ANN in polyphenol extraction modeling.

When the complexity of the extraction system exceeds the descriptive capability of linear or quadratic relationships—such as in multi-technology synergy or multi-objective optimization—Artificial Neural Networks (ANN) demonstrate unique value. The core advantage of ANN is its powerful non-linear fitting capability, enabling it to capture complex response trends in high-dimensional parameter spaces, often achieving higher prediction accuracy than RSM (Figure 12) [94].

However, this advantage comes at the cost of “interpretability”: ANN is a typical “black box” model, where internal weights are difficult to map directly to physical or chemical mechanisms. This contrasts sharply with RSM, which is based on explicit mathematical forms (polynomial equations) where coefficients directly relate to process variable effects. Thus, ANN and RSM form a complementary relationship: ANN excels at high-performance prediction and pattern discovery in broad, mechanism-opaque parameter spaces, while RSM is suitable for fine-grained parameter analysis and window optimization in local spaces where mechanisms are clearer. Evidence from large-scale, cross-system studies in heterogeneous environmental matrices further suggests that nonlinear, data-driven models become most actionable when anchored to interpretable mechanistic features, supporting the rationale for combining predictive learning with explainable, mechanism-informed optimization [95,96]. Together, they enable a “Prediction–Explanation–Re-optimization” loop. For instance, in scale-up scenarios, ANN can update models using online or near-online data to respond to process fluctuations in real-time [97,98].

In summary, RSM and ANN should not be viewed as mutually exclusive. Their value is maximized when integrated into a framework based on molecular interactions, structural disruption, and mass-transfer control. To further demonstrate the practical application of these tools in complex scenarios, the following section discusses specific cases of multi-objective optimization in collaborative extraction systems.

### 6.4. Multi-Objective Optimization Cases in Collaborative Systems

In synergistic extraction systems such as DES-UAE, the interdependencies among input variables generate highly nonlinear response surfaces. For example, while increasing ultrasonic power can enhance polyphenol yield by disrupting cell walls, excessive power may simultaneously induce oxidative degradation and sharply increase energy consumption. Therefore, single-factor optimization approaches exhibit significant limitations. A typical multi-objective optimization scenario involves balancing three key indicators: yield, purity (selectivity), and energy efficiency.

Application of Response Surface Methodology (RSM): A Box–Behnken design was used to arrange combinations of key parameters (water content, vortex time, and liquid-to-solid ratio). This generated a structured dataset to capture interactions and supported subsequent ANN training.

Integration of Artificial Neural Networks (ANN): When nonlinearity exceeded the descriptive capacity of RSM, a backpropagation-based artificial neural network (BP-ANN) was adopted to improve nonlinear prediction. The network featured a 3-9-1 topology, comprising three input neurons, nine hidden layer neurons, and one output neuron. During training, the experimental dataset generated in the prior RSM phase was used and partitioned into training, validation, and test sets at ratios of 70%, 15%, and 15%, respectively. Optimized via the Levenberg–Marquardt algorithm and aimed at minimizing mean squared error (MSE), the network ultimately constructed a predictive model capable of accurately capturing the complex nonlinear relationships between input variables and total flavonoid extraction yield.

Pareto Optimization: During the parameter optimization stage, the trained ANN predictive model served as the objective function, and a genetic algorithm (GA) was utilized to perform global optimization across the high-dimensional nonlinear response surface constructed by the ANN, thereby identifying the optimal extraction conditions. Within defined parameter constraints (e.g., water content: 20–40%, vortex time: 8–12 min, liquid-to-solid ratio: 20–40 mL/g), the algorithm iteratively searched and ultimately output a precise set of optimal parameters: water content of 31.75%, vortex time of 10.33 min, and liquid-to-solid ratio of 34.12 mL/g, corresponding to a predicted extraction yield of 26.36 mg/g. For practical implementation, these parameters were appropriately adjusted to water content of 32%, vortex time of 10 min, and liquid-to-solid ratio of 34:1 mL/g, providing a practical and executable process window for subsequent experimental validation.

Experimental Verification: Subsequent experiments conducted under the optimized conditions yielded an extraction amount of 26.39 ± 0.61 mg/g, demonstrating high consistency with the predicted value. The relative error (0.12%) and RSD (0.24%) of the RSM-ANN-GA model were both lower than those of the traditional RSM model, indicating superior accuracy and stability [99].

Beyond static optimization, Table 6 illustrates the transition of green extraction toward ‘Intelligent Collaboration’ and Industry 4.0. Unlike previous method-oriented reviews that focus solely on descriptive modeling, this framework emphasizes adaptive decision-making. By integrating *Adaptive Control* and multi-objective Pareto optimization, researchers can now balance the inherent trade-offs between extraction efficiency and energy footprint. This shift reflects industrial realities, where matrix heterogeneity and sensor-based feedback often limit process sustainability.

## 7. Conclusions and Future Perspectives

The efficient and green extraction of plant polyphenols has transitioned from an era dominated by empirical solvent screening to a multi-scale system defined by the interplay of “molecular interactions—tissue architecture—mass transfer processes.” From a mechanism-driven perspective, this review has systematically elucidated how structural heterogeneity, the coexistence of free/bound states, and interactions with cell wall components (e.g., hydrogen bonding, hydrophobic effects, and π–π stacking) constitute the molecular basis of extractability. We identify cell wall integrity and internal diffusion as key rate-limiting factors, and integrate the mechanistic roles of conventional solvents, green solvents (DESs, SFE), and physical-field intensification.

Looking forward, to address the core concerns of plant science and sustainable engineering, future research must prioritize the following directions:

(1) Unlocking “Plant-Side Determinants” for Targeted Extraction: Research should move beyond generic parameters and quantify raw-material variability. Critical questions remain regarding how different species, genotypes, and tissue types (e.g., peel vs. seed) dictate the spatial distribution of polyphenols within subcellular compartments (vacuoles vs. cell walls). Key needs include resolving the binding-energy barriers between bound phenolics and the polysaccharide–lignin network. This knowledge will support “targeted extraction”—the rational design of green solvent and energy field combinations that specifically match the tissue structure and target subclass to balance yield, selectivity, and stability.
(2) From Passive Optimization to Intelligent Decision-Making: As extraction systems increase in complexity, process optimization is shifting from being a supporting step to a decision-enabling module. While Response Surface Methodology (RSM) remains valid for identifying robust operating windows under mechanistic constraints, data-driven models like Artificial Neural Networks (ANN) are indispensable for handling non-linear coupling and multi-objective predictions. Integrating mechanistic features with RSM/ANN can support scale-up and stable operation.
(3) Bridging the Gap to Industrialization: Industrial translation must adhere to the principles of “reproducibility, recyclability, and regulatability.” Priority should be given to continuous, modular, and integrated flows that narrow the lab-to-industry gap. Furthermore, incorporating Life Cycle Assessment (LCA), safety evaluations, and regulatory compliance into the early design phase—combined with digital twins and intelligent control—will drive polyphenol extraction from being merely “lab-optimal” to being “scalable, compliant, and sustainable”.

**Figure 13:**
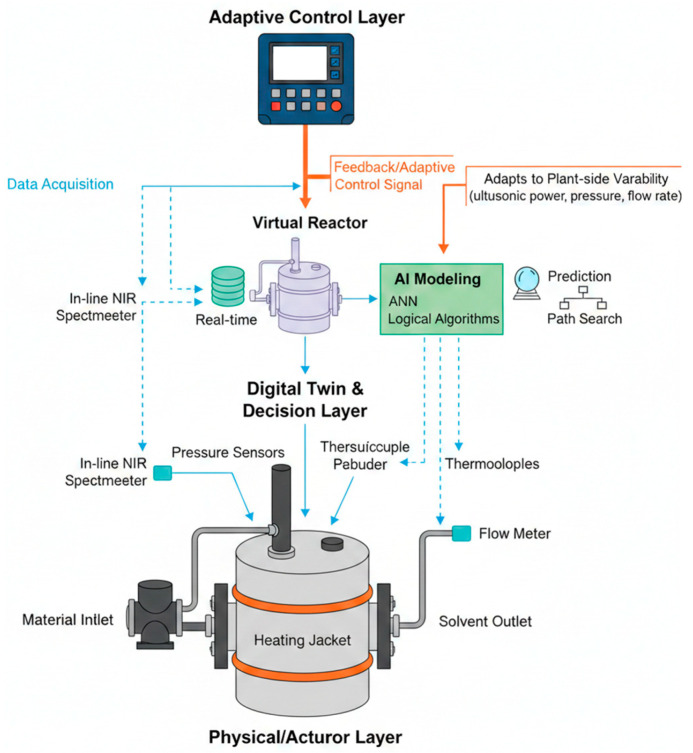
Proposed architecture for an Intelligent Extraction System (IES) integrating mechanism-driven insights with Industry technologies.

To translate lab optima into industrial practice, we propose an Intelligent Extraction System (IES) architecture (as illustrated in Figure 13). The framework comprises: (1) Physical/Actuator Layer for synergistic energy/solvent input; (2) Sensing Layer for real-time monitoring of process variables (e.g., NIR, T, P); (3) Decision Layer utilizing Digital Twins and ANN models for predictive analysis; and (4) Adaptive Control Layer that facilitates autonomous process adjustments to ensure optimal bioactivity retention and energy efficiency across heterogeneous plant matrices. The core is a Digital Twin (DT) that mirrors the extraction process. By deploying a multi-sensor network (e.g., in-line NIR for polyphenol concentration and ultrasonic power transducers), real-time data is fed into a database. AI models then drive adaptive control to compensate for biomass variability.

In conclusion, we present a “mechanism-driven, synergistic integration” framework. By decoding the molecular interactions within plant matrices and strategically coupling complementary technologies, we transcend the limitations of empirical optimization. This framework supports both mechanistic design in the lab and intelligent, sustainable implementation at scale.

## Figures and Tables

**Table 1:** Correlation between structural characteristics of major plant polyphenol classes and their extraction behavior.

Polyphenol Class	Representative Chemical Forms	MW Range (Da) & Polarity Trend	Dominant Matrix Interaction & Energy Barrier	Primary Rate-Limiting Step & pKa Influence	Critical Stability Issues & Literature Evidence	Ref.
Flavonoids (e.g., Quercetin, Rutin)	Aglycones (lipophilic); O/C-glycosides (hydrophilic)	250–600+: MW increases with sugar moieties. Aglycones: low polarity; Glycosides: high polarity	Hydrogen bonding: Glycosides interact with cell wall hemicellulose; Aglycones often compartmentalized in cuticular waxes	Dissolution/Diffusion: pKa\approx 6.5–9.5. Solubility increases in alkaline media but risks ring-opening	Glycosidic bonds prone to hydrolysis (<pH 2 or >pH 10); thermo-oxidative degradation during extended reflux	[16,17,18]
Phenolic Acids (e.g., Ferulic, Caffeic)	Free forms; Soluble esters; Insoluble bound forms	150–400: Generally low MW. Polarity is high due to terminal carboxyl (-COOH) groups	Covalent bonding: Ester/ether links to lignin and arabinoxylans	Chemical Dissociation: Release depends on bond cleavage (pH or enzymatic) rather than simple diffusion	Carboxyl groups sensitive to decarboxylation; esters easily hydrolyzed in alkaline conditions	[19,20]
Anthocyanins	Monomeric glycosides; Acylated anthocyanins	400–1000: MW highly variable depending on acylation (e.g., caffeic/p-coumaric acid)	Ionic/H-bonding: Interaction with negatively charged pectins; highly soluble in vacuoles	Equilibrium-limited leaching: Limited by the flavylium cation stability (pH < 3.0)	Extreme pH sensitivity: Structural transformation to colorless chalcones at neutral pH; rapid thermal browning	[21,22,23]
Tannins (Condensed/Hydrolyzable)	Oligomers; High-degree polymers (DP > 10)	500–3000+: Extremely high MW and density of phenolic hydroxyls	Hydrophobic π–π stacking: Strong multi-point binding to proteins and cell wall lignin	Desorption/Macromolecular Transfer: Slow diffusion due to large hydrodynamic radius and matrix entrapment	Easily form insoluble precipitates with salivary proteins; prone to irreversible oxidative condensation	[24,25,26]

**Table 2:** Comparison of green extraction technologies for polyphenols: Mechanistic suitability based on chemical speciation and matrix complexity.

Technology	Fundamental Mechanism	Target Chemical Speciation	Matrix-Specific Advantages	Primary Constraints	Ref.
DESs	Disrupts cell wall-polyphenol interactions by competing for hydrogen bonds.	Strongly hydrogen-bonded polyphenols (e.g., flavonoids, tannins)	It readily penetrates complex plant matrices (e.g., lignocellulosic structures) to effectively extract cell wall-embedded polyphenols.	High viscosity restricts mass transfer and complicates subsequent separation and purification steps.	[29,30,31,32,33]
SFE	Supercritical CO_2_ as a tunable-polarity solvent	Low-MW Aglycones; Lipophilic phenolics (e.g., specific flavonoids)	Excellent for thermolabile compounds; Oxygen-free environment prevents oxidation.	Limited for high-polarity glycosides without excessive co-solvent.	[34,35,36,37]
UAE	Acoustic cavitation and mechanical shear	Vacuolar polyphenols; Intracellular glycosides and free acids	Efficiently disrupts soft plant tissues; Enhances mass transfer of large molecules.	Potential degradation of sensitive anthocyanin glycosides via hot spots.	[38,39,40,41]
MAE	Dipolar rotation and ionic conduction	Monomeric & Oligomeric phenolics; Thermostable glycosides	Rapid penetration of moist matrices; Effective for deep-seated metabolites.	High risk of glycoside hydrolysis and degradation of p-coumaric derivatives.	[42,43,44,45]
EAE	Enzymatic hydrolysis of cell-wall polymers	Cell-wall-bound forms; Ester-linked phenolic acids	Targets covalent bonds (ester/ether) that physical methods cannot break.	Long incubation times; Specificity limited by enzyme–substrate fit.	[46,47]
HHPE	Causes irreversible physical damage to plant cell structures,	Completely extract and preserve various polyphenolic compounds (e.g., flavonoids, phenolic acids)	Hard, dense, or thicker-walled plant materials	For polyphenols that are covalently bound or located within specific organelles, the extraction efficiency is lower.	[48,49]
PLE/PHWE	Subcritical water/solvent under pressure	Broad-spectrum; Particularly high-MW tannins & glycosides	High pressure forces solvent into dense lignocellulosic structures.	Risk of Maillard reactions and structural rearrangement at high T.	[50,51]

**Table 4:** Strategic optimization of synergistic extraction parameters based on polyphenol speciation and matrix resistance.

Parameter	Synergy Mechanism	Response of Different Chemical Speciation	Optimal Window Logic	Critical Risks (Side Reactions)	Ref.
Solvent Polarity	Polarity matching with target solutes	Aglycones: Requires low Δ (SFE/Hexane); Glycosides: High Δ(Ethanol/Water).	Matching the Hansen solubility parameters of specific sugar moieties.	Phase separation or excessive co-extraction of sugars/pigments.	[80,81]
Temperature	Enhances kinetic energy and solubility	Esterified forms: T promotes bond cleavage; Anthocyanins: Highly T-sensitive.	Balancing the activation energy of diffusion vs. the degradation kinetics.	Hydrolysis of glycosidic bonds and Maillard browning.	[82,83,84]
Ultrasonic Power	Cavitation-induced structural disruption	Bound phenolics: High P breaks cell-wall entrapment; Tannins: Enhances desorption.	Maintaining P above the cavitation threshold but below the radical-generation level.	Radical-induced oxidation and chain scission of high-MW polymers.	[63,85,86]
Enzyme Activity	Catalytic cleavage of matrix linkages	Insoluble bound acids: Highly dependent on cellulase/pectinase activity.	Optimizing the enzyme-to-substrate ratio for specific cell-wall architectures.	Non-specific hydrolysis affecting the structural integrity of glycosides.	[46]
Solid–Liquid Ratio	Driving force for concentration gradient	All forms: High S/L increases the concentration gradient but raises cost.	Ensuring the saturation limit is not reached for low-solubility aglycones.	Mass transfer stagnation due to high viscosity (especially in DESs systems).	[87,88,89]

**Table 5:** Case studies of RSM and ANN modeling in synergistic extraction: Capturing non-linear interactions across polyphenol speciation.

Source/Matrix	Target Speciation & Molecular Feature	Technology	Model	Key Interactions & Non-Linearity Captured	Optimization Outcome	Ref.
Pomelo peel	Hydrolyzable tannins (High MW, H-bond rich)	UAE-EAE	RSM/ANN	Interaction between enzyme loading and ultrasonic intensity; captured the threshold for tannin-protein complex dissociation.	25% increase in ellagic acid yield vs. single EAE.	[91]
Citrus peel	Flavanone glycosides (e.g., Hesperidin; Thermosensitive)	MAE	RSM	Non-linear effect of microwave power on glycosidic bond stability; modeled the “micro-explosion” window for cell-wall rupture.	Optimized yield at 450 W with minimal oxidative degradation.	[87]
Jujube	Flavonoids (primarily rutin, with a total of 15 compounds identified)	DES-UAE	RSM	The stability of flavonoids is strongly influenced by both extraction conditions (heat/light exposure) and storage parameters (temperature, light, duration).	An enhanced DES-UAE protocol results in flavonoids with optimal stability.	[92]
Madras thorn peel	Bound phenolic acids (Covalently linked)	MAE	RSM/ANN-GA	The ANN-GA model accurately predicts the non-linear effects of microwave parameters on phenolics and bioactivities (higher R^2^).	The ANN-GA model demonstrates excellent predictive accuracy (R^2^ 0.9805–0.9813) alongside low statistical error.	[93]

**Table 6:** Advanced machine learning and intelligent frameworks in green extraction: From laboratory optimization to industrial adaptive control.

System/Algorithm	Targeted Functional Feature	Application Scenario	Intelligent Breakthrough & Decision Logic	Implementation Challenges Identified	Ref.
ANN-GA Hybrid	Multi-objective Pareto optimization	UAE of complex matrices	Balances extraction yield against energy consumption; captured the “sweet spot” of ultrasonic intensity.	High computational cost for real-time recalibration.	[100]
Deep Learning (CNN)	Image-based matrix characterization	Dynamic monitoring of cell disruption	Visualizes structural deconstruction in real-time; correlates surface morphology with mass transfer rates.	Requires massive labeled datasets of plant micro-structures.	[101]
Adaptive Control (PID-AI)	Real-time parameter adjustment	Continuous flow extraction systems	Automatically adjusts flow rates based on in-line NIR monitoring of polyphenol concentration.	Sensor durability in high-pressure or cavitation environments.	[102]

## Data Availability

The data that support the findings of this study are available from the corresponding author upon reasonable request.
